# Evaluation of standard of care intravitreal aflibercept treatment of diabetic macular oedema treatment-naive patients in the UK: DRAKO study 12-month outcomes

**DOI:** 10.1038/s41433-021-01624-9

**Published:** 2021-07-09

**Authors:** Sobha Sivaprasad, Faruque Ghanchi, Simon P. Kelly, Ajay Kotagiri, James Talks, Peter Scanlon, Hellen McGoey, Andrew Nolan, Moneeb Saddiq, Jackie Napier, on behalf of the DRAKO study group

**Affiliations:** 1 National Institute for Health Research, Moorfields Biomedical Research Centre, London, UK; 2 Bradford Teaching Hospitals NHS Foundation Trust, Bradford, UK; 3 Bolton Hospital NHS Foundation Trust, Bolton, UK; 4 South Tyneside and Sunderland NHS Foundation Trust, Sunderland, UK; 5 Newcastle Upon Tyne Hospitals NHS Foundation Trust, Newcastle Upon Tyne, UK; 6 Gloucestershire Hospitals NHS Foundation Trust, Cheltenham, UK; 7 Bayer Plc, Reading, UK; 8 O4 Research Limited, Belfast, UK

**Keywords:** Diseases, Signs and symptoms

## Abstract

**Objectives:**

DRAKO (NCT02850263) is a 24-month, prospective, non-interventional, multi-centre cohort study which enroled patients diagnosed with centre-involving diabetic macular oedema (DMO). The study aims to evaluate standard of care with intravitreal aflibercept (IVT-AFL) treatment in the UK. This analysis describes the anti-vascular endothelial growth factor (anti-VEGF) treatment-naive patient cohort after 12-month follow-up.

**Methods:**

Study eyes were treated with IVT-AFL as per local standard of care. The mean change in best-corrected visual acuity (BCVA) and central subfield thickness (CST) from baseline at 12 months were measured and stratified by baseline factors. The number of injections and safety data were also evaluated.

**Results:**

A total of 507 patients were enroled from 35 centres. Mean (SD) baseline BCVA was 71.4 (12.0) letters and CST was 448.7 (88.7) µm, with 63.1% of patients presenting with baseline BCVA ≥ 70 letters (mean 78.1). Mean (SD) change in BCVA of 2.5 (12.2) letters and CST of −119.1 (116.4) µm was observed at month 12. A 7.3 letter gain was observed in patients with baseline BCVA < 70 letters. Mean number (SD) of injections in year one was 6.4 (2.1). No significant adverse events were recorded.

**Conclusion:**

Year one results indicated that IVT-AFL was an effective treatment for DMO in standard of care UK clinical practice, maintaining or improving visual acuity in treatment-naive patients with good baseline visual acuity, despite some patients being undertreated versus the summary of product characteristics. These results also demonstrated the clinical importance and meaningful impact of diabetic retinopathy screening in the UK.

## Introduction

Diabetes, including its associated complications, is an escalating healthcare problem, with treatment estimated to cost around 10% of the UK’s entire National Health Service (NHS) budget [1]. The global prevalence of diabetes is projected to increase from the current 9.3% to 10.2% in 2030, affecting 578.4 million adults between the ages of 20–79 years, further increasing the burden on health systems [2]. Diabetic macular oedema (DMO), a microvascular complication of diabetes that can occur at any stage of retinopathy, is the most common cause of visual acuity (VA) loss in patients with diabetes and the most frequent cause of blindness in young and mid-aged adults in the developed world. One in four diabetic patients can expect to develop DMO in their lifetime [3–6].

In recent years, the use of anti-vascular endothelial growth factor (anti-VEGF) treatments for DMO has seen outcomes for many patients improve. Intravitreal injections of anti-VEGF therapies have shown superiority over laser photocoagulation of the macula in several clinical trials [7–10]. Subsequently, intravitreal VEGF inhibitors have become the first-line therapy of choice for management of vision loss from DMO.

Intravitreal aflibercept (IVT-AFL, Eylea) is an anti-VEGF treatment with an innovative fusion protein design. Current IVT-AFL standard of care treatment for DMO patients is a 2 mg dose of five initial monthly injections followed by a proactive bi-monthly treatment regimen in year one, with no requirement for monitoring between injections. In year two, the injection interval may be extended dependent on functional and anatomical outcomes, consequently reducing burden on patients and their care providers (IVT-AFL summary of product characteristics [SmPC] recommended posology for DMO treatment) [11].

Results from the two pivotal IVT-AFL Phase 3 trials in DMO, VIVID^DMO^ and VISTA^DMO^, demonstrated significant superiority for the IVT-AFL treatment groups over laser in all endpoints at week 52 through to week 148, with similar efficacy in both treatment regimens [10, 12, 13].

The UK did not participate in these pivotal trials, and DRAKO represents the first UK-based prospective, non-interventional study to assess the effectiveness of standard of care IVT-AFL treatment in DMO patients. Such observational studies are valuable as they report and evaluate outcomes based on locally defined treatment practices, outside the rigorous clinical trial setting, enabling outcome characterisation within a more representative population and treatment environment.

The primary objectives of this study are to assess the mean change from baseline in best-corrected visual acuity (BCVA) and central subfield thickness (CST). In addition, by evaluating follow-up procedures and assessing treatment patterns in eyes affected by DMO treated with IVT-AFL in routine clinical practice, the study aims to inform best practice guidance in the UK upon completion of the follow-up period. Here we describe the month 12 outcomes of the anti-VEGF treatment-naive patient cohort.

## Materials and methods

### Study design

DRAKO (NCT02850263) is a prospective, non-interventional, multi-centre, non-comparative cohort study conducted in 35 NHS hospitals throughout the UK (Supplementary Table 1). Patients were enroled from July 2016 through to April 2018 and followed up for 24 months. To reduce selection bias, patients were enroled in a consecutive manner. The study was approved by the North West Liverpool East Research Ethics Committee (16/NW/0238) and was conducted in accordance with the Declaration of Helsinki. All participants provided written informed consent. All treatment decisions, including the decision to treat with IVT-AFL, were made as per local standard of care, independently of study participation. The study included two patient cohorts; Cohort 1 comprised anti-VEGF treatment-naive patients and Cohort 2 comprised patients who previously received anti-VEGF treatment other than IVT-AFL for DMO. Study size was calculated based on an assumed 10% drop-out rate at 24 months, allowing an estimate of change from baseline in BCVA letters within 95% confidence intervals (CI) of ±1.2 letters and CST of ±13.9 μm. This was based on a standard deviation (SD) of 13 letters for the mean change in BCVA and 150 µm for CST, conservative estimates based on SD observed in other recent studies involving aflibercept in DMO [10, 14]. Sample sizes of 450 and 225 were calculated for Cohorts 1 and 2 respectively.

### Study population and treatment

Adult patients with a confirmed diagnosis of type 1 or type 2 diabetes and DMO with central involvement, defined as the centre subfield area on optical coherence tomography, were enroled if they met the eligibility criteria as defined in Table 1. Inclusion of patients based on a CST of ≥400 µm in the study eye was removed as an eligibility criterion via protocol amendment (21 February 2017) as this threshold is not applied in Scotland and removal enabled a more accurate representation of the UK-treated population. One of the two participating Scottish sites (University Hospital Hairmyres) was active prior to approval of the protocol amendment. The study eye was defined as the eye for which the IVT-AFL treatment was initiated or, if both eyes were affected, the eye with worse baseline VA. Data were collected for the fellow eye if DMO diagnosis was confirmed. No eligibility restriction was mandated for patient baseline BCVA letter score.

**Table 1:** Patient inclusion and exclusion criteria based on cohort for the DRAKO non-interventional study.

**Inclusion criteria**	
*Universal study inclusion criteria*	*Anti-VEGF treatment-naive cohort specific criteria*
Aged 18 years or older	Not previously received anti-VEGF treatment
Type 1 or 2 diabetes mellitus diagnosis	Not received pan-retinal photocoagulation treatment in the past 8 weeks
	Not received macular laser photocoagulation treatment in the past 4 months
DMO diagnosis with central involvement	Not previously received intravitreal dexamethasone steroid or fluocinolone acetonide treatment
	*Prior Anti-VEGF treatment other than IVT-AFL cohort specific criteria*
Decision to initiate IVT-AFL treatment made as per routine practice and before study inclusion	Previously treated with an intravitreal anti-VEGF other than aflibercept for DMO
	Not received intravitreal anti-VEGF treatment in the past 28 days
Written informed consent	Not received intravitreal dexamethasone steroid treatment in the past 6 months
**Exclusion criteria**	
Contraindications as listed in the SmPC for IVT-AFL	
Pre-planned cataract surgery during the observational period	
Currently or previously treated with systemic anti-VEGF	
Previously treated with intravitreal fluocinolone acetonide steroid	
Participating in an investigational programme with interventions outside of routine clinical practice	
Women who are currently pregnant or lactating and/or planning pregnancy within the next 2 years	
Known hypersensitivity to any excipients	
Active or suspected ocular/periocular infection or periocular inflammation	

*DMO* diabetic macular oedema, *IVT-AFL* intravitreal aflibercept, *anti-VEGF* anti-vascular endothelial growth factor, *SmPC* summary of product characteristics.

At study initiation, details of the local standard of care IVT-AFL treatment protocol was recorded for each centre (Supplementary Table 2). Data collection, including type of visit; diabetic management (diabetic retinopathy measured using the English National Screening Committee or Scottish Diabetic Retinopathy Grading Scheme classifications, and haemoglobin A_1c_ (HbA_1c_) assessment), anatomical and functional assessments; treatment provided from the baseline visit; and all subsequent routine outpatient visits were recorded. The month 12 visit was nominated by the site (for data collection purposes) and defined as 12 months ±1 month from the patient’s baseline visit.

Patients who dropped out of the study for any reason were not replaced.

### Outcome measures

Primary outcomes were the mean change from baseline in Early Treatment Diabetic Retinopathy Study (ETDRS) letters measured by BCVA with refraction and the mean change in CST as determined by spectral domain optical coherence tomography (as per local practice) at month 12. Secondary objectives included mean change in BCVA and CST stratified by pre-defined baseline factors; percentage gain and loss of at least 5, 10 or 15 letters; and adherence to IVT-AFL DMO SmPC in year one. Due to the observational design of the study, target changes in these parameters were not defined.

### Statistical analysis

Interim analysis was conducted upon completion of the 12-month follow-up period for the treatment-naive patient cohort and are reported here.

Descriptive statistics were used to summarise the quantitative variables. Categorical variables were summarised using frequency distributions and percentages and data stratified by pre-defined baseline covariate subgroups; age (18–35, 36–50, 51–65, >65 years), BCVA (<35, 35–49, 50–69, ≥70 letters) and CST (<400, ≥400 µm).

All patients with a baseline IVT-AFL injection and at least one post-baseline assessment of BCVA or CST were included in the analysis. Two sub-populations were analysed, and 95% CI were calculated. The more stringent sub-population, defined as the per protocol window population (PPW), included patients with available BCVA or CST data at baseline and the nominated month 12 visit (12 months ± 1 month). The less stringent sub-population was defined as the full analysis set (FAS) and included patients with BCVA or CST available at baseline and at least one follow-up visit; missing values were imputed based on the last observation carried forward method, the visit immediately preceding the month 12 visit was used for analysis. Adherence to SmPC was measured by defining acceptable ‘windows’ between injections. For the first five injections, it was 25–38 days; after the fifth injection, it was 46–66 days.

Safety was assessed on the safety set, which included all patients who provided written informed consent. Adverse events were listed using the Medical Dictionary for Regulatory Activities coding system.

Analysis was performed using SAS^®^ software, version 9.4 (SAS Institute Inc., Cary, NC, USA).

## Results

### Patient disposition and baseline characteristics

**Fig. 1:**
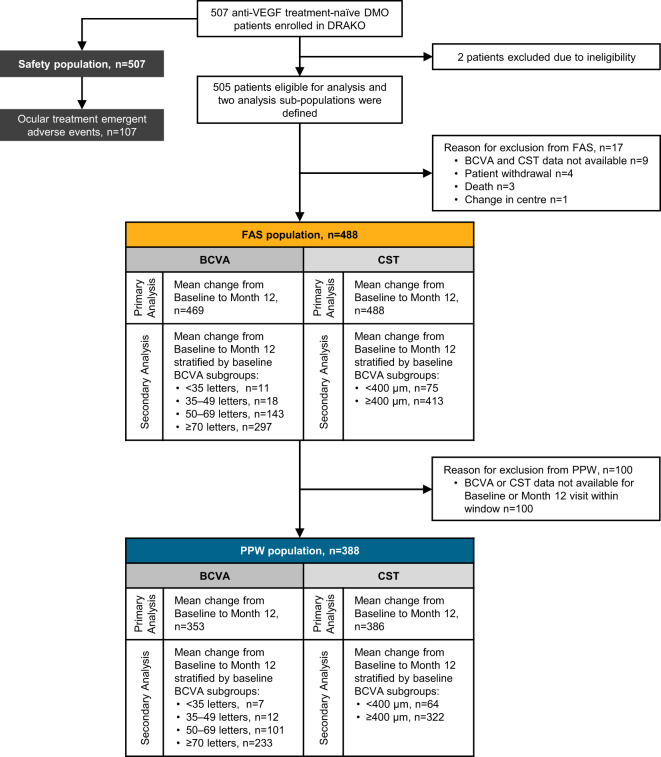
Patient disposition during the study and analysis summary. DMO diabetic macular oedema, anti-VEGF anti-vascular endothelial growth factor, IVT-AFL intravitreal aflibercept, PPW per protocol window population, FAS full analysis set, BCVA best-corrected visual acuity, CST central subfield thickness, n number of patients per group.

DRAKO enroled 507 anti-VEGF treatment-naive patients. A 2.0% drop-out rate was observed in year one, the reasons for drop-out were as follows; patient withdrawal (4), death (3), patient ineligibility (2) and change in centre (1). Further patients were excluded from the analysis due to unavailability of baseline and/or 12-month data. Patients were divided into PPW (*n* = 388) and FAS (*n* = 488) sub-populations for analysis based on pre-defined stringency criteria (Fig. 1). The PPW and FAS sub-populations demonstrated comparable trends throughout the analysis and results for the PPW sub-population are reported unless stated otherwise.

The mean age at baseline was 62.9 years, and 63.1% of patients were male (Table 2). Most patients were white (76.8%), and 85.8% had been diagnosed with type 2 diabetes. Over half of patients had fellow eye involvement. Patients demonstrated a suboptimal level of glycaemic control with mean (SD) HbA_1c_ of 66.1 (20.5) mmol/mol. Background to pre-proliferative retinopathy was recorded for the majority of patients regardless of classification applied. On average, patients had been diagnosed with DMO 1.3 (SD 2.4) years before their baseline visit. Of the primary endpoint assessments, BCVA was recorded for 91.0% of patients and CST for 99.5% of patients. The mean (SD) baseline BCVA and CST were 71.4 (12.0) letters and 448.7 (88.7) µm, respectively (Table 3).

**Table 2:** Patient demographics and baseline characteristics per sub-population.

	PPW	FAS
Age, years, mean (SD)		
All	62.9 (11.6)	62.4 (11.6)
18–35	30 (4.3)	29.4 (4.4)
36–50	45.2 (3.5)	45.6 (3.4)
51–65	58.9 (4.3)	58.7 (4.2)
>65	73.4 (5.9)	73.3 (5.8)
Sex, *n* (%)		
Male	245 (63.1)	303 (62.1)
Female	143 (36.9)	185 (37.9)
Race or ethnicity, *n* (%)		
White	298 (76.8)	374 (76.6)
Asian	38 (9.8)	50 (10.2)
Black	29 (7.5)	38 (7.8)
Mixed	4 (1.0)	4 (0.8)
Hispanic	2 (0.5)	3 (0.6)
Unknown	14 (3.6)	15 (3.1)
Other	3 (0.8)	4 (0.8)
Diabetes type, *n* (%)		
1	55 (14.2)	67 (13.7)
2	333 (85.8)	421 (86.3)
Diabetic retinopathy, *n* (%)		
*English Scale*	265 (85.8)	340 (87.4)
R0	4 (1.5)	5 (1.5)
R1	111 (41.9)	142 (41.8)
R2	104 (39.2)	133 (39.1)
R3	46 (17.4)	60 (17.6)
*Scottish Scale*	44 (14.2)	49 (12.6)
R0	0	0
R1	21 (47.7)	25 (51.0)
R2	21 (47.7)	21 (42.9)
R3	2 (4.5)	3 (6.1)
R4	0	0
HbA1c (mmol/ mol), *n* mean (SD)		
*n*	194	239
Mean (SD)	66.1 (20.5)	66.2 (20.3)
Fellow eye involvement, *n* (%)		
DMO confirmed in fellow eye	209 (53.9)	265 (54.3)
Diabetes diagnosis to baseline, mean (SD) years	16.0 (10.5)	15.8 (10.3)
DMO diagnosis to baseline, years, mean (SD)	1.3 (2.4)	1.3 (2.4)

*SD* standard deviation, *n* number of patients, *PPW* per protocol window population, *FAS* full analysis set, *DMO* diabetic macular oedema.

**Table 3:** Mean change from baseline in functional and anatomical endpoints at month 12.

Assessment	Per protocol window (*n* = 388)			Full analysis set (*n* = 488)		
	Mean (SD)	95% CI	Patient number	Mean (SD)	95% CI	Patient number
BCVA						
Baseline	71.4 (12.0)	NA	375	70.8 (12.7)	NA	469
Month 12	73.6 (13.9)	NA	362	72.5 (14.3)	NA	481
Change from baseline	2.5 (12.2)	1.3, 3.8	353	1.9 (11.3)	0.9, 2.9	469
CST						
Baseline	448.7 (88.7)	NA	388	452.1 (87.2)	NA	488
Month 12	329.8 (92.3)	NA	386	334.4 (94.1)	NA	488
Change from baseline	–119.1 (116.4)	–130.7, –107.4	386	–117.7 (113.7)	–127.8, –107.6	488

Baseline and Month 12 mean (SD) for the primary endpoints with patient number defined per analysis sub-population. Mean change in BCVA and CST from Baseline were reported, and 95% CI were calculated.

*SD* standard deviation, *BCVA* best-corrected visual acuity, *CST* central subfield thickness, *CI* confidence interval, *NA* not applicable.

### Treatment of DMO

Local standard of care IVT-AFL treatment was administered across all sites, with 11 different treatment posologies recorded at study initiation (Supplementary Table 2). All patients received at least one IVT-AFL injection in the study eye. The mean number (SD) of injections was 6.4 (2.1) and 6.3 (2.2) for the PPW and FAS sub-populations, respectively, compared with eight to nine injections if treatment was conducted as per the SmPC. At site initiation, 27 (77.1%) and 21 (60.0%) of the 35 participating sites confirmed their intention to adhere to DMO SmPC for the five initial monthly injections and for 12 months respectively as per their local standard of care protocol. This equated to 334 patients (86.1%) from the 27 sites intending to administer five initial injections and 211 (54.4%) from the 21 sites intending to adhere to SmPC throughout year one. However, in practice, 117 (30.2%) of patients received five initial IVT-AFL injections and 13 (3.4%) patients adhered to SmPC in year one.

### Effect of treatment on functional and anatomical outcomes

**Fig. 2:**
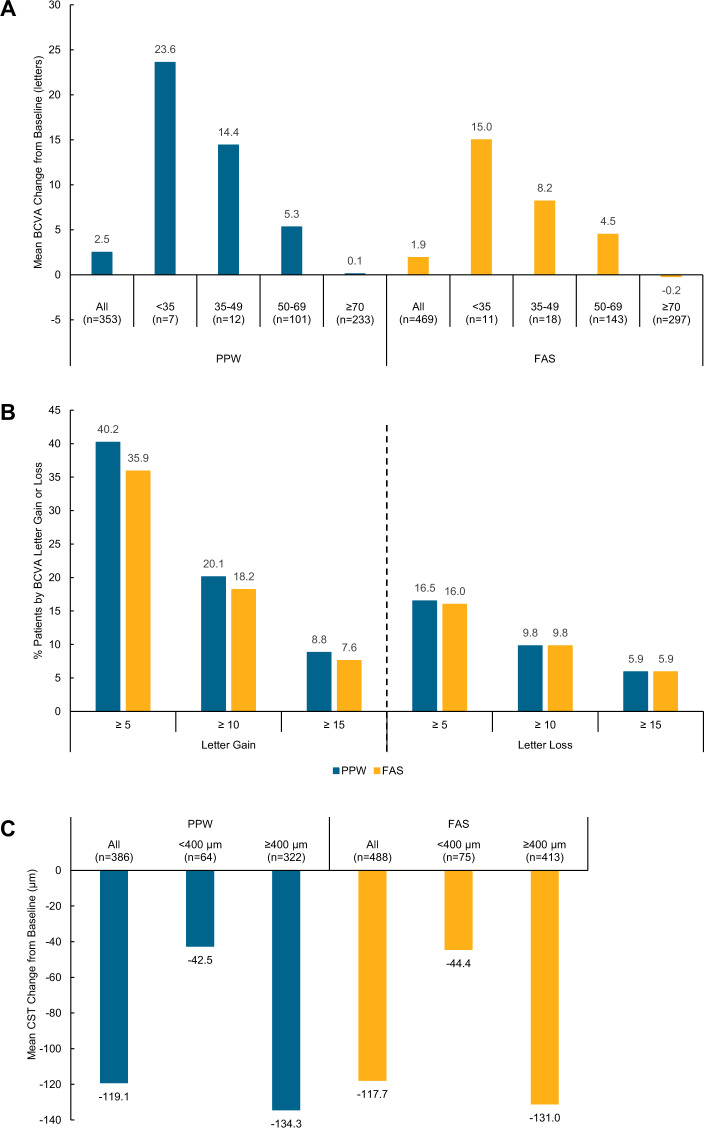
Primary outcome results stratified by baseline measure and BCVA letter gain or loss proportion. **A** BCVA stratification, **B** BCVA letter gain and loss proportion and **C** CST stratification, by sub-population. BCVA best-corrected visual acuity, CST central subfield thickness, PPW per protocol window population, FAS full analysis set.

The mean (95% CI) improvement in BCVA at month 12 was 2.5 letters (±1.3 letters) and 1.9 letters (±1.0 letters) for the PPW and FAS sub-populations, respectively (Table 3). The relative increase in BCVA letters varied based on initial VA. Patients with baseline BCVA of less than 35 letters benefitted from a mean increase of 23.6 letters (*n* = 7), whereas the mean BCVA for patients presenting with BCVA of 70 letters or more remained stable (Fig. 2A and Supplementary Table 3). Similarly, patients aged 18–35 years experienced a larger increase in mean BCVA letters than those aged 65 years and older (4.3 and 1.2 letters, respectively; Supplementary Table 3). A letter gain of five or more was observed in 40.2% of patients, whereas an equivalent letter loss was noted in 16.5% patients (Fig. 2B). When evaluated by adherence to SmPC, patients receiving five initial monthly injections obtained a mean (SD) letter gain of 4.2 (10.9). The small proportion of patients for whom treatment continued as per SmPC in year one experienced a 1.1 (7.4) letter gain (Supplementary Table 4).

At month 12, CST was reduced, with mean (95% CI) change from baseline determined as –119.1 µm (±11.6 µm) and –117.7 µm (±10.1 µm) for the PPW and FAS sub-populations, respectively (Table 3). Baseline CST was <400 µm for 16.5% of patients. Mean (SD) CST change was observed as –42.5 (76.3) µm and –134.3 (117.0) µm for patients with initial CST measure of <400 µm and ≥400 µm, respectively (Fig. 2C and Supplementary Table 5). A larger improvement in BCVA was observed at month 12 for patients presenting with a baseline CST of ≥400 µm (mean [SD] BCVA letter change was 1.4 [9.2] and 2.7 [12.6] for patients with CST of <400 µm and ≥400 µm, respectively; see Supplementary Table 3).

### Adverse events

The safety set comprised data from the full treatment-naive patient cohort (*n* = 507). During the first 12 months of treatment, a total of 3129 injections were administered to the study eye. Non-ocular treatment emergent adverse events (TEAE) were reported for 33.7% (*n* = 171) of patients and ocular TEAEs for 13.2% (*n* = 67) of patients. A total of 107 ocular TEAEs were captured. Reported TEAEs (percentage of patients) included vitreous haemorrhage (3.0%), cataract (1.2%), retinal haemorrhage (1.2%), retinal detachment (0.2%) and vitreous detachment (0.2%). Adverse events reported as associated with treatment were primarily related to the procedure rather than aflibercept. The safety profile was in line with other published studies, and no new safety concerns were identified. Further details on reported TEAEs are outlined in Supplementary Table 6.

## Discussion

DRAKO is the first prospective, multi-centre study evaluating standard of care IVT-AFL treatment for DMO in the UK. The study enroled 507 treatment-naive patients across 35 contributing centres and it was noted that the majority of patients with DMO are diagnosed and treated at a high level of VA, demonstrating the effectiveness of the diabetic retinopathy screening programme in the UK.

The mean baseline BCVA for DRAKO (71.4 letters) was substantially higher than measures reported in pivotal clinical trials (VIVID^DMO^ and VISTA^DMO^ aflibercept cohorts reported mean baseline BCVA of 59.8 letters and 59.1 letters, respectively) and in other prospective observational DMO studies (APOLLON and POLARIS) [10, 15, 16]. Of note, two-thirds of DRAKO patients had baseline BCVA of 70 letters or more (mean 78.1). APOLLON, a French IVT-AFL treatment-based study enroled 77 patients between September 2016 and July 2019, reported mean baseline VA 62.7 letters in the treatment-naive cohort [15]. However, eligibility was limited to patients with baseline VA ≤20/40 (≤70 ETDRS letters). POLARIS, a European-based, ranibizumab study that recruited 125 patients within the UK cohort from September 2012 to January 2015 reported a mean baseline VA of 60.3 letters [16]. When comparing baseline VA in these UK cohorts, a clinically relevant difference of 11 letters in baseline VA was observed between DRAKO and POLARIS. Although these letter changes are not directly comparable given that DRAKO captured BCVA with refraction and POLARIS documented non-refracted VA, these measures are nonetheless highly correlated. This may be a further indication of a shift towards earlier screening and treatment of DMO within the NHS in more recent years, particularly as DRAKO observed a mean time from diagnosis of DMO to baseline treatment of 1.3 years, suggesting that patients are often being diagnosed with DMO in advance of the condition being considered clinically significant. Indeed, these results suggest that the National Institute for Health and Care Excellence (NICE) Technical Appraisal Guidance on the threshold for treatment initiation (central retinal thickness of at least 400 µm) may be encouraging early intervention to maintain or improve patient VA in areas of the UK where these guidelines apply (England, Wales and Northern Ireland).

DRAKO patients experienced a mean gain of 2.5 letters and a mean reduction in CST of 119.1 µm at month 12. Trends in letter gains and losses demonstrated that 40.2% and 20.1% patients observed at least a 5- or 10-letter gain respectively within the first year. Whereas, 16.5% and 9.8% patients observed at least a 5- or 10-letter loss respectively within the same period. A 5-letter gain in the context of the high observed baseline BCVA scores may be clinically relevant in most patients. In addition, stratification by baseline BCVA demonstrated that better gains were achieved in those with worse baseline BCVA. Of note, for patients with a baseline BCVA ≤ 69 letters (mean 58.7 letters), the mean gain at month 12 was 7.3 letters.

Although these trends reflect those seen in both IVT-AFL treatment randomised clinical trials (VIVID^DMO^ and VISTA^DMO^) and the APOLLON observational study, the overall 12-month BCVA letter gains observed in these studies are higher than in DRAKO (gain of 10.7, 10.7 and 7.8 letters, respectively) [10, 15]. This variation is likely due to the protocol-driven higher injection numbers, particularly in the pivotal trials (patients received five initial monthly doses followed by IVT-AFL treatment every 8 weeks) and the increased opportunity for larger BCVA gains from a lower baseline as a result of implementing a maximum BCVA inclusion criteria which was not mandated for DRAKO.

Previous reports have highlighted the positive association of injection frequency and functional outcomes for patients, with an emphasis on the initial injections [17]. The DRAKO study authors also advocate the administration of full initial dosing, with the subgroup of patients receiving five initial monthly injections obtaining a mean letter gain of 4.2, although this benefit was not extended to year one SmPC compliance, potentially due to the low patient numbers or high baseline BCVA. The reasons for the lower than intended number of injections administered were not collected and changes in standard of care treatment protocols at participating sites were not re-assessed after site initiation. Consequently, it was not possible to determine whether this was due primarily to capacity constraints within the NHS or other factors, such as clinical prioritisation, patient influences or perhaps a more relaxed treatment approach in response to favourable baseline metrics. Of note, APOLLON results showed a higher rate of patients receiving five initial doses of IVT-AFL (51.9%) than DRAKO, which may also have contributed to the higher letter gain reported.

Overall, DRAKO patients with a baseline BCVA of ≥70 letters maintained their vision. Those with baseline BCVA of 70–78 letters (*n* = 139) gained 1.3 letters, and those with baseline BCVA > 78 (*n* = 94) experienced a loss of 1.8 letters. This distinction may be important in efforts to preserve ‘driving vision’, which is categorised in the UK as a BCVA of 70 letters or more in the patient’s better eye. The results for patients with BCVA 78 letters or more are comparable to those of the Diabetic Retinopathy Clinical Research Retina Network Protocol V study. Whilst no control group was included in the DRAKO study design, the reduced benefits observed in this subgroup support the conclusion of Protocol V that a reasonable strategy for patients with high VA may be observation without treatment unless VA worsens, although there were few patients with >400 µm CST in Protocol V (9% patients in the aflibercept group) [18]. Analysis of the DRAKO year 2 results may provide further clarity on this clinically important point.

These DRAKO observations are supported by several retrospective registry studies, which demonstrated the real-world effectiveness of IVT-AFL for DMO treatment [19–21]. Notably, fewer injections were recorded compared to clinical trials, and functional gains were not as pronounced and were related to baseline measures. In the UK-based, single-centre retrospective cohort study conducted by Lukic et al. [21]., 99 eyes were evaluated and a mean of 6.9 injections were administered. The baseline VA (59.7 ETDRS letters) in this study was much lower than DRAKO, so although the overall letter gain (9.9 letters) was higher than that observed in DRAKO, in the 34% of eyes with a baseline VA ≥69, the mean gain was 2.6 letters.

A similar ‘ceiling’ effect was observed in the CST subgroup analysis. Baseline CST was stratified above or below 400 µm to align with the NICE Technology Appraisal Guidance. The reduction in CST for the baseline ≥400 µm subgroup was much greater than for the <400 µm subgroup, but the mean CST at month 12 was similar for both subgroups. The results suggest minimal difference in terms of BCVA outcomes between the two subgroups, with a marginally lower letter gain attained in the <400 µm subgroup, although patient numbers were limited (*n* = 52).

The study has some limitations often inherent in non-interventional studies such as, inconsistent treatment administration and non-defined functional eligibility metrics. However, the prospective study design and wide range of sites contributing enables treatment effects to be monitored across a diverse population that aims to be representative of the UK as a whole. In addition, the large number of anti-VEGF treatment-naive patients enroled, and the high proportion of primary endpoint data collected, strengthen the observations made during the study.

In conclusion, year one results indicate that IVT-AFL is an effective treatment for DMO in real-world UK clinical practice, maintaining or improving VA. Vision gains observed in DRAKO were lower than in the pivotal clinical trials and several observational studies [10, 14–16], perhaps reflecting better baseline vision in UK clinical practice and/or a failure to adhere to the SmPC for all patients. In addition, DRAKO demonstrated the effectiveness of diabetic retinopathy screening in the UK, with almost two-thirds of patients presenting with good baseline vision.

The DRAKO data set contains a rich source of real-world ocular-, treatment- and resource-related data that will mature further upon completion of the study. It is anticipated that this will provide a benchmark for better understanding of DMO IVT-AFL outcomes and for shaping how DMO patient care pathways are defined in the future.

## Summary

### What was known before

- The effectiveness of intravitreal aflibercept (IVT-AFL) for treatment of diabetic macular oedema (DMO) patients has been demonstrated in several pivotal clinical trials (VIVID and VISTA) and non-UK focused observational studies (APOLLON), although such investigations primarily focused on patients with baseline visual acuity of <73 letters.
- Retrospective registry-based studies of anti-vascular endothelial growth factor (anti-VEGF) treatments have reported lower injection frequency and functional gains than randomised clinical trials.
- The scope for improvement of functional and anatomical parameters in response to anti-VEGF treatment is closely associated with baseline values.

### What this study adds

- DRAKO is the first prospective non-interventional study to evaluate IVT-AFL treatment of DMO patients across the UK, demonstrating the effectiveness of this treatment to maintain or improve patient outcomes across a diverse range of local standard of care protocols, despite often observing undertreatment compared with locally defined treatment plans.
- DRAKO indicates the effectiveness of the diabetic retinopathy screening programme in the UK where patients with DMO are being identified and treated at a high level of VA, thereby preserving patient vision.

## Supplementary information

Supplementary files summary

Supplementary Table 1

Supplementary Table 2

Supplementary Table 3

Supplementary Table 4

Supplementary Table 5

Supplementary Table 6
