# Supplementary material for: Evaluation of standard of care intravitreal aflibercept treatment of diabetic macular oedema treatment-naive patients in the UK: DRAKO study 12-month outcomes
Source: Eye (Lond). 2021 Jul 9;36(1):64–71. doi: 10.1038/s41433-021-01624-9 (PMC8727562; doi:10.1038/s41433-021-01624-9)
Supplement: Supplementary file 1 — Supplementary files summary [file 41433_2021_1624_MOESM1_ESM.docx]

Supplementary Data Files

Supplementary Table 1

List of DRAKO investigative sites and associated principal investigators.

File type: .docx

Supplementary Table 2

Local standard of care protocol intravitreal aflibercept treatment intent at DRAKO study initiation in descending order based on SmPC compliance.

File type: .docx

Supplementary Table 3

Mean (SD) results for change in best corrected visual acuity (BCVA) at month 12, stratified by age and baseline BCVA and central subfield thickness for both per protocol window (PPW) and full analysis (FAS) set sub-populations.

File type: .docx

Supplementary Table 4

Mean change from baseline in functional and anatomical endpoints at month 12 based on SmPC treatment compliance.

File type: .docx

Supplementary Table 5

Mean (SD) results for change in central subfield thickness (CST) at month 12, stratified by age and baseline best corrected visual acuity and CST for both per protocol window (PPW) and full analysis set (FAS) sub-populations.

File type: .docx

Supplementary Table 6

Treatment emergent adverse events (TEAEs) reported for the DRAKO safety population as categorised by MedDRA Systems Organ Classes preferred term. Number and percentage of patients and number of events per term are defined. Eye disorders (ocular) TEAEs have been listed in full.

File type: .docx
