# Supplementary material for: Evaluation of standard of care intravitreal aflibercept treatment of diabetic macular oedema treatment-naive patients in the UK: DRAKO study 12-month outcomes
Source: Eye (Lond). 2021 Jul 9;36(1):64–71. doi: 10.1038/s41433-021-01624-9 (PMC8727562; doi:10.1038/s41433-021-01624-9)
Supplement: Supplementary file 2 — Supplementary Table 1 [file 41433_2021_1624_MOESM2_ESM.docx]

Supplementary Table 1. List of DRAKO investigative sites and associated principal investigators.

| **Site Name** | **Principal Investigator** |
| --- | --- |
| Aintree University Hospital | Ms Nihal Kenaway |
| Altnagelvin Area Hospital | Mr Asif Orakzai |
| Barnet Hospital | Mr Hemal Mehta  Mr Martin Harris |
| Bradford Royal Infirmary | Professor Faruque Ghanchi |
| Bristol Eye Hospital | Ms Clare Bailey |
| Central Middlesex Hospital | Ms Christiana Dinah |
| Essex County Hospital | Mr Jignesh Patel |
| Frimley Park Hospital | Professor Geeta Menon |
| Gloucestershire Hospitals NHS Foundation Trust | Professor Peter Scanlon |
| James Cook University Hospital | Mr Philip Severn |
| James Paget University Hospital | Professor Ben Burton |
| Kent and Canterbury Hospital | Mr Afsar Jafree |
| King's College Hospital | Mr Haralabos Eleftheriadis |
| Leicester Royal Infirmary | Mr Vasileious Konidaris |
| Manchester Royal Eye Hospital | Mr Sajjad Mahmood  Professor Tariq Aslam |
| Moorfields Eye Hospital | Professor Sobha Sivaprasad  (Chief Investigator) |
| Norfolk and Norwich University Hospital | Mr Colin Jones |
| Queen Alexandra Hospital | Ms Sarah Meredith |
| Royal Bolton Hospital | Mr Simon Kelly |
| Royal Victoria Infirmary | Mr James Talks |
| Salisbury District Hospital | Ms Rashi Arora |
| Sandwell General Hospital | Ms Bushra Mushtaq |
| Singleton Hospital | Mr Gwyn Williams |
| Southampton General Hospital | Professor Andrew Lotery |
| St James's University Hospital | Mr Martin McKibbin |
| Sunderland Eye Infirmary | Mr Ajay Kotagiri |
| The Princess Alexandra Hospital | Ms Priyah Prakash |
| The Royal Liverpool University Hospital | Ms Amira Stylianides  Professor Simon Peter Harding |
| The Royal Free Hospital | Mr Riaz Asaria |
| University Hospital Ayr | Mr Mohan Varikkara |
| University Hospital Hairmyres | Ms Meena Virdi |
| University Hospital of Wales | Mr Sanjiv Banerjee |
| Western Eye Hospital | Mr Saad Younis |
| Westmorland General Hospital | Mr Simon Morgan |
| York Hospital | Ms Nicola Topping  Professor Richard Gale |
