# Supplementary material for: Evaluation of standard of care intravitreal aflibercept treatment of diabetic macular oedema treatment-naive patients in the UK: DRAKO study 12-month outcomes
Source: Eye (Lond). 2021 Jul 9;36(1):64–71. doi: 10.1038/s41433-021-01624-9 (PMC8727562; doi:10.1038/s41433-021-01624-9)
Supplement: Supplementary file 3 — Supplementary Table 2 [file 41433_2021_1624_MOESM3_ESM.docx]

Supplementary Table 2. Local standard of care protocol IVT-AFL treatment intent at DRAKO study initiation in descending order based on SmPC compliance.

| **Number of different protocols** | **Local standard of care protocol for IVT-AFL treatment of DMO** | **Sites adhering to each local standard of care protocol**  **n (%)** | **Total number of PPW patients enrolled by centres based on defined treatment intent** |
| --- | --- | --- | --- |
| 1 | SmPC compliant – 5 monthly injections, then bimonthly injections in year 1 | 21 (60.0) | 211 |
| 2 | 5 monthly injections, PRN | 2 (5.7) | 41 |
| 3 | 5 monthly injections, then treat and extend | 2 (5.7) | 50 |
| 4 | 5 monthly injections, then if dry patients are monitored, if still wet additional 3 monthly injection. If dry but becomes wet, three 2 monthly injections | 1 (2.9) | 7 |
| 5 | 5 monthly injections, followed by adjuvant focal laser, if appropriate or bi-monthly injection up to month 12, if good response to initial dosing | 1 (2.9) | 25 |
| 6 | 3-5 monthly injections, then follow SmPC guidelines | 1 (2.9) | 3 |
| 7 | Monthly injections, if stable then treat and extend | 1 (2.9) | 5 |
| 8 | 4 consecutive monthly doses then PRN | 1 (2.9) | 4 |
| 9 | 3 monthly injections then PRN | 3 (8.6) | 18 |
| 10 | 3 monthly injection given based on baseline VA of 6/12 or worse. Review and repeat as required. | 1 (2.9) | 11 |
| 11 | 3 monthly injections, then assessment in nurse led clinic including further 3 monthly injections. After 6 injections patient followed-up in a doctor led clinic. | 1 (2.9) | 13 |
| Total number of potential patients being treated as per IVT-AFL SmPC in year one based on local standard of care intention at study initiation = 211 (Protocol 1) | | | |
| Total number of potential patients receiving 5 initial monthly injections of IVT-AFL based on local standard of care intention at study initiation = 334 (Protocols 1 – 5) | | | |
| IVT-AFL = Intravitreal aflibercept; PPW = Per protocol window; SmPC = Summary of Product Characteristics; PRN = pro re nata/ taken as needed; VA = visual acuity. | | | |
