# Supplementary material for: Evaluation of standard of care intravitreal aflibercept treatment of diabetic macular oedema treatment-naive patients in the UK: DRAKO study 12-month outcomes
Source: Eye (Lond). 2021 Jul 9;36(1):64–71. doi: 10.1038/s41433-021-01624-9 (PMC8727562; doi:10.1038/s41433-021-01624-9)
Supplement: Supplementary file 4 — Supplementary Table 3 [file 41433_2021_1624_MOESM4_ESM.docx]

Supplementary Table 3. Mean (SD) results for change in BCVA at month 12, stratified by baseline factors.

| **BCVA Stratified by Baseline Factors** | | | **PPW (n=388)** | | | **FAS (n=488)** | | |
| --- | --- | --- | --- | --- | --- | --- | --- | --- |
|  |  |  | **Baseline** | **12 Months** | **Change from Baseline** | **Baseline** | **12 Months** | **Change from Baseline** |
| **Age (years) at Baseline Subgroup** | **18-35** | **n** | 9 | 9 | 9 | 11 | 12 | 11 |
|  |  | **Mean (SD)** | 69.0 (21.4) | 73.3 (15.0) | 4.3  (12.4) | 70.9 (19.8) | 75.8 (13.6) | 4.1  (11.8) |
|  | **36-50** | **n** | 40 | 35 | 35 | 52 | 52 | 52 |
|  |  | **Mean (SD)** | 73.0 (12.7) | 77.7 (11.9) | 4.2  (10.6) | 72.3 (12.9) | 75.4 (12.7) | 3.1  (8.9) |
|  | **51-65** | **n** | 169 | 165 | 159 | 219 | 226 | 219 |
|  |  | **Mean (SD)** | 72.0 (12.3) | 74.5 (15.3) | 3.3  (13.4) | 71.1 (13.0) | 73.1 (15.4) | 2.4  (12.5) |
|  | **>65** | **n** | 157 | 153 | 150 | 187 | 191 | 187 |
|  |  | **Mean (SD)** | 70.4 (10.8) | 71.8 (12.4) | 1.2  (11.0) | 70.0 (11.8) | 70.9 (13.2) | 0.9  (10.4) |
| **BCVA Letters at Baseline Subgroup** | **<35** | **n** | 7 | 16 | 7 | 11 | 23 | 11 |
|  |  | **Mean (SD)** | 28.7 (4.7) | 58.0 (20.8) | 23.6  (13.9) | 29.4  (4.5) | 56.0 (22.3) | 15.0 (16.0) |
|  | **35-49** | **n** | 12 | 12 | 12 | 18 | 18 | 18 |
|  |  | **Mean (SD)** | 43.3 (4.3) | 57.7 (16.1) | 14.4  (15.6) | 42.2  (4.8) | 50.4 (17.4) | 8.2  (16.4) |
|  | **50-69** | **n** | 111 | 101 | 101 | 143 | 143 | 143 |
|  |  | **Mean (SD)** | 62.3 (5.3) | 67.6 (12.0) | 5.3  (11.4) | 62.1  (5.3) | 66.6 (11.4) | 4.5  (10.8) |
|  | **≥70** | **n** | 245 | 233 | 233 | 297 | 297 | 297 |
|  |  | **Mean (SD)** | 78.1 (5.8) | 78.1 (11.5) | 0.1  (11.1) | 78.2  (5.9) | 78.0 (10.7) | -0.2 (10.2) |
| **CST µm at Baseline Subgroup** | **<400 µm** | **n** | 60 | 55 | 52 | 70 | 74 | 70 |
|  |  | **Mean (SD)** | 72.8 (13.8) | 72.9 (16.5) | 1.4  (9.2) | 73.5 (13.4) | 73.1 (15.1) | 0.9  (8.4) |
|  | **≥400 µm** | **n** | 315 | 307 | 301 | 399 | 407 | 399 |
|  |  | **Mean (SD)** | 71.1 (11.6) | 73.8 (13.4) | 2.7  (12.6) | 70.3 (12.5) | 72.4 (14.1) | 2.1  (11.7) |
| BCVA = best-corrected visual acuity; PPW = per protocol window population; FAS = full analysis set; CST = central subfield thickness, n = number of patients; SD = standard deviation. | | | | | | | | |
