# Supplementary material for: Evaluation of standard of care intravitreal aflibercept treatment of diabetic macular oedema treatment-naive patients in the UK: DRAKO study 12-month outcomes
Source: Eye (Lond). 2021 Jul 9;36(1):64–71. doi: 10.1038/s41433-021-01624-9 (PMC8727562; doi:10.1038/s41433-021-01624-9)
Supplement: Supplementary file 5 — Supplementary Table 4 [file 41433_2021_1624_MOESM5_ESM.docx]

Supplementary Table 4. Mean change from Baseline in functional and anatomical endpoints at Month 12 based on SmPC treatment compliance in the Per Protocol Window population.

|  | **Received 5 initial monthly injections within window** | | **Adhered to SmPC in Year 1** | |
| --- | --- | --- | --- | --- |
| **Assessment** | **Mean (SD)** | **Patient Number** | **Mean (SD)** | **Patient Number** |
| **BCVA** | | | | |
| Baseline | 70.6 (12.1) | 111 | 72.2 (10.3) | 13 |
| Month 12 | 74.3 (11.7) | 108 | 72.9 (11.8) | 12 |
| Change from Baseline | 4.2 (10.9) | 104 | 1.1 (7.4) | 12 |
| **CST** | | | | |
| Baseline | 443.3 (79.9) | 117 | 409.2 (80.0) | 13 |
| Month 12 | 335.1 (100.0) | 116 | 304.7 (86.4) | 13 |
| Change from Baseline | -108.5 (110.0) | 116 | -104.5 (77.3) | 13 |
| Mean (SD) Baseline, Month 12 and change from Baseline at Month 12 for BCVA and CST based on administration of either 5 initial monthly IVT-AFL injections or SmPC compliance in Year one (8–9 IVT-AFL injections).  IVT-AFL = intravitreal aflibercept; SmPC = Summary of Product Characteristics; SD = standard deviation; BCVA = best corrected visual acuity; CST = central subfield thickness. | | | | |
