# Supplementary material for: Evaluation of standard of care intravitreal aflibercept treatment of diabetic macular oedema treatment-naive patients in the UK: DRAKO study 12-month outcomes
Source: Eye (Lond). 2021 Jul 9;36(1):64–71. doi: 10.1038/s41433-021-01624-9 (PMC8727562; doi:10.1038/s41433-021-01624-9)
Supplement: Supplementary file 6 — Supplementary Table 5 [file 41433_2021_1624_MOESM6_ESM.docx]

Supplementary Table 5. Mean (SD) results for change in CST at month 12, stratified by baseline factors.

| **CST Stratified by Baseline Factors** | | | **PPW (n=388)** | | | **FAS (n=488)** | | |
| --- | --- | --- | --- | --- | --- | --- | --- | --- |
|  |  |  | **Baseline** | **12 Months** | **Change from Baseline** | **Baseline** | **12 Months** | **Change from Baseline** |
| **Age (years) at Baseline Subgroup** | **18-35** | **n** | 9 | 9 | 9 | 12 | 12 | 12 |
|  |  | **Mean (SD)** | 477.1 (92.8) | 275.0 (63.2) | –202.1 (134.3) | 459.0 (90.4) | 288.0 (60.7) | –171.0 (128.6) |
|  | **36-50** | **n** | 40 | 40 | 40 | 52 | 52 | 52 |
|  |  | **Mean (SD)** | 429.8 (84.9) | 334.5 (104.6) | –95.2 (111.1) | 441.4 (86.4) | 330.2 (101.8) | –111.1 (114.6) |
|  | **51-65** | **n** | 177 | 177 | 177 | 231 | 231 | 231 |
|  |  | **Mean (SD)** | 458.7 (97.0) | 331.4 (85.8) | –127.4 (127.1) | 459.8 (92.9) | 336.7 (92.4) | –123.2 (122.9) |
|  | **>65** | **n** | 162 | 160 | 160 | 193 | 193 | 193 |
|  |  | **Mean (SD)** | 440.9 (78.2) | 329.9 (97.1) | –111.1 (101.4) | 445.4 (79.7) | 335.7 (95.6) | –109.7 (99.9) |
| **BCVA Letters at Baseline Subgroup** | **<35** | **n** | 7 | 7 | 7 | 11 | 11 | 11 |
|  |  | **Mean (SD)** | 523.7 (112.5) | 353.6 (118.4) | –170.1 (187.6) | 506.6 (92.1) | 335.8 (96.8) | –170.8 (148.6) |
|  | **35-49** | **n** | 12 | 12 | 12 | 18 | 18 | 18 |
|  |  | **Mean (SD)** | 480.3 (117.9) | 326.8 (77.0) | –153.4 (147.5) | 508.6 (115.5) | 342.2 (105.6) | –166.4 (148.3) |
|  | **50-69** | **n** | 111 | 111 | 111 | 143 | 143 | 143 |
|  |  | **Mean (SD)** | 465.0 (87.2) | 333.3 (101.8) | –131.8 (121.1) | 467.4 (87.0) | 334.0 (104.0) | –133.4 (117.2) |
|  | **≥70** | **n** | 245 | 243 | 243 | 297 | 297 | 297 |
|  |  | **Mean (SD)** | 436.5 (68.7) | 326.4 (87.4) | –110.1 (94.3) | 438.2 (68.7) | 332.1 (88.4) | –106.1 (94.6) |
| **CST µm at Baseline Subgroup** | **<400 µm** | **n** | 64 | 64 | 64 | 75 | 75 | 75 |
|  |  | **Mean (SD)** | 339.3 (50.2) | 296.8 (74.1) | –42.5 (76.3) | 341.3 (47.5) | 296.8 (71.2) | –44.4 (74.7) |
|  | **≥400 µm** | **n** | 324 | 322 | 322 | 413 | 413 | 413 |
|  |  | **Mean (SD)** | 470.3 (78.0) | 336.3 (94.3) | –134.3 (117.0) | 472.2 (77.1) | 341.2 (96.2) | –131.0 (114.6) |
| BCVA = best-corrected visual acuity; PPW = per protocol window population; FAS = full analysis set; CST = central subfield thickness, n = number of patients; SD = standard deviation. | | | | | | | | |
