# Supplementary material for: Evaluation of standard of care intravitreal aflibercept treatment of diabetic macular oedema treatment-naive patients in the UK: DRAKO study 12-month outcomes
Source: Eye (Lond). 2021 Jul 9;36(1):64–71. doi: 10.1038/s41433-021-01624-9 (PMC8727562; doi:10.1038/s41433-021-01624-9)
Supplement: Supplementary file 7 — Supplementary Table 6 [file 41433_2021_1624_MOESM7_ESM.docx]

Supplementary Table 6. Treatment emergent adverse events (TEAEs) categorised by MedDRA System Organ Class preferred term. Number and percentage of patients and number of events per term are defined. Eye disorders (ocular) TEAEs have been listed in full.

| **System Organ Class Preferred Term** | **Patients, n (%)** | **TEAEs, n** |
| --- | --- | --- |
| *Any Treatment Emergent Adverse Event (TEAE)* | *193 (38.1)* | *580* |
|  |  |  |
| *Ocular TEAEs – eye disorders* | *67 (13.2)* | *107* |
| Blepharitis | 4 (0.8) | 4 |
| Cataract | 6 (1.2) | 7 |
| Chalazion | 1 (0.2) | 1 |
| Conjunctival haemorrhage | 2 (0.4) | 2 |
| Diabetic keratopathy | 1 (0.2) | 1 |
| Diabetic retinal edema | 1 (0.2) | 1 |
| Diabetic retinopathy | 1 (0.2) | 1 |
| Dry eye | 3 (0.6) | 3 |
| Erythema of eyelid | 1 (0.2) | 1 |
| Eye discharge | 2 (0.4) | 2 |
| Eye pain | 4 (0.8) | 4 |
| Eyelid pain | 1 (0.2) | 1 |
| Foreign body sensation in eyes | 3 (0.6) | 3 |
| Glaucoma | 1 (0.2) | 1 |
| Iris neovascularisation | 3 (0.6) | 3 |
| Lacrimation increased | 4 (0.8) | 5 |
| Macular fibrosis | 2 (0.4) | 2 |
| Macular hole | 1 (0.2) | 1 |
| Narrow anterior chamber angle | 1 (0.2) | 1 |
| Ocular hyperaemia | 5 (1.0) | 5 |
| Photophobia | 1 (0.2) | 1 |
| Posterior capsule opacification | 1 (0.2) | 1 |
| Retinal detachment | 1 (0.2) | 1 |
| Retinal haemorrhage | 6 (1.2) | 8 |
| Retinal neovascularisation | 2 (0.4) | 2 |
| Retinal tear | 2 (0.4) | 2 |
| Retinal vein occlusion | 4 (0.8) | 4 |
| Swelling of eyelid | 3 (0.6) | 3 |
| Vision blurred | 5 (1.0) | 5 |
| Visual impairment | 5 (1.0) | 5 |
| Vitreous adhesions | 3 (0.6) | 3 |
| Vitreous detachment | 1 (0.2) | 1 |
| Vitreous floaters | 3 (0.6) | 3 |
| Vitreous haemorrhage | 15 (3.0) | 19 |
|  |  |  |
| *Non-ocular TEAE* | 171 (33.7) | 473 |
| Blood and lymphatic system disorders | 6 (1.2) | 9 |
| Cardiac disorders | 31 (6.1) | 38 |
| Corneal perforation | 1 (0.2) | 1 |
| Diabetic ketoacidosis | 1 (0.2) | 2 |
| Ear and labyrinth disorders | 1 (0.2) | 2 |
| Endocrine disorders | 1 (0.2) | 1 |
| Gastrointestinal disorders | 22 (4.3) | 30 |
| General disorders and administration site conditions | 21 (4.1) | 28 |
| Hepatobiliary disorders | 2 (0.4) | 3 |
| Immune system disorders | 2 (0.4) | 2 |
| Infections and infestations | 61 (12.0) | 87 |
| Injury, poisoning and procedural complications | 29 (5.7) | 42 |
| Investigations | 20 (3.9) | 25 |
| Metabolism and nutrition disorders | 12 (2.4) | 20 |
| Musculoskeletal and connective tissue disorders | 11 (2.2) | 12 |
| Neoplasms benign, malignant and unspecified (including cysts and polyps) | 9 (1.8) | 10 |
| Nervous system disorders | 31 (6.1) | 42 |
| Product issues | 1 (0.2) | 2 |
| Psychiatric disorders | 6 (1.2) | 6 |
| Renal and urinary disorders | 15 (3.0) | 23 |
| Reproductive system and breast disorders | 3 (0.6) | 4 |
| Respiratory, thoracic and mediastinal disorders | 17 (3.4) | 23 |
| Sepsis | 1 (0.2) | 1 |
| Skin and subcutaneous tissue disorders | 23 (4.5) | 27 |
| Surgical and medical procedures | 14 (2.8) | 22 |
| Vascular disorders | 11 (2.2) | 11 |
| TEAEs = treatment-emergent adverse events; MedDRA = Medical Dictionary for Regulatory Activities; SOC = system organ class. | | |
